# Rapid assessment of ocular drug delivery in a novel ex vivo corneal model

**DOI:** 10.1038/s41598-020-68254-1

**Published:** 2020-07-16

**Authors:** Ghazala Begum, Thomas Leigh, Ella Courtie, Richard Moakes, Gibran Butt, Zubair Ahmed, Saaeha Rauz, Ann Logan, Richard J. Blanch

**Affiliations:** 1 miRNA Diagnostics, Birmingham, UK; 2 Neuroscience and Ophthalmology, Institute of Inflammation and Ageing, University of Birmingham, Birmingham, UK; 3 NIHR Surgical Reconstruction and Microbiology Research Centre, University of Birmingham, Birmingham, UK; 4 School of Chemistry, University of Birmingham, Birmingham, UK; 5 School of Chemical Engineering, University of Birmingham, Birmingham, UK; 6 Academic Unit of Ophthalmology, Birmingham and Midland Eye Centre, University of Birmingham, Birmingham, UK; 7 Academic Department of Military Surgery and Trauma, Royal Centre for Defence Medicine, Birmingham, UK; 8 Department of Ophthalmology, University Hospitals Birmingham NHS Foundation Trust, Birmingham, UK

**Keywords:** Biological techniques, Drug discovery

## Abstract

Drug delivery by topical application has higher patient acceptance and lower morbidity than intraocular injection, but many ophthalmic treatments are unable to enter the eye or reach the posterior segment after topical application. The first stage towards posterior segment delivery after topical application is ocular surface penetration and existing models are in vivo or use large quantities of tissue. We therefore developed a novel ex vivo model using discs of porcine and human cornea and sclera (5 mm diameter) to assess penetration of a candidate neuroprotective siRNA. siRNA against caspase 2 or control solutions of known penetrance were applied to the corneal epithelial surface and trans-corneal penetration and corneal adsorbance measured at fixed time points. To demonstrate that leakage did not occur, we applied dextran blue, which should not penetrate the intact cornea and did not do so in our model. Fluorescein penetration (0.09%) was less than rhodamine B (6.98%) at 60 min. siCASP2 penetration was 0.01% by 60 min. When the applied siCASP2 was washed off after 2 min, (representing lacrimal drainage) 0.071% penetrated porcine cornea by 60 min and 0.0002% penetrated human cornea and 0.001% penetrated human sclera. Our ex vivo model rapidly and cost-effectively assesses transcorneal penetration of candidate topical therapies, allowing rates of trans-corneal penetration for potential therapies such as siRNA to be evaluated with small quantities of human or animal tissue.

## Introduction

Ocular penetration of topically-applied drugs remains a pharmaceutical challenge. Small interfering ribonucleic acid (siRNA) molecules have the potential to treat a wide range of ophthalmic pathologies^1^, generating an interest in agents to enhance their penetration after topical application^2^. In particular, siRNA against caspase-2 (siCASP2) prevents retinal ganglion cell death and is in Phase 3 clinical trials for non-arteritic anterior ischaemic optic neuropathy (NCT02341560; ClinicalTrials.gov)^3,4^. Current application of siRNA requires intraocular injection, which is invasive, carries a risk of infection and is less acceptable to patients than other methods of drug delivery.

Diffusion of compounds through the eye after topical application is hindered by the presence of anatomical barriers including the tear film, cornea, conjunctiva, sclera, choroid, aqueous, lens and vitreous^5^. Having passed through these barriers, compounds are additionally cleared by vascular or aqueous drainage^5,6^. The blood-aqueous and the blood-retinal barriers provide challenges for systemic drug delivery in addition to systemic toxicity^7–9^. As a result, after initial topical application approximately 1/100,000 of compounds such as steroids including prednisolone acetate will reach the back of the eye^5,10^. The therapeutic efficacy of such dilutions may not be sufficient for biologically relevant treatment effects. However, topical delivery offers the potential for non-invasive treatment of intra-ocular diseases with greater patient acceptance and reduced complications compared to intraocular injection.

Topically applied compounds may enter the eye by two routes: trans-corneal or trans-scleral. The conjunctiva overlying sclera has a greater pore size than the cornea at 3.0 nm ± 1.6; however, the underlying blood and lymphatic vessels clear topically administered drugs^11^. Whilst the cornea provides a more direct route of absorption, the pore diameter is much lower at 2.0 nm ± 0.2 limiting penetration to compounds with a molecular weight of < 500Da^10,11^. Drug-penetration is also dependent on lipophilicity, because lipophilic drugs may penetrate through transcellular pathways. Hydrophilic compounds must cross the cornea by the paracellular route^5^, which is blocked by corneal epithelial tight junctions between cells preventing hydrophilic drug passage.

Models for the assessment of ocular drug delivery include in vitro, ex vivo and more commonly used in vivo models. Rabbits have been routinely used for in vivo modelling of drug delivery, as rabbits are readily available for research, have extensive ocular pharmacokinetic data for comparison and have eyes of comparable but smaller size compared to human eyes, although corneal thickness is 150 µm lower in rabbits and there are species differences in drug transporter expression^12–14^. In contrast pig corneal thickness is comparable to peripheral human corneal thickness but with little variation between central and peripheral locations^15^. The use of these larger animals in vivo such as pigs and monkeys are significantly more costly, whilst smaller animals such as rats and mice have eyes significantly structurally different to humans.

Ex vivo models reported include rabbit, porcine and bovine whole corneas in Franz diffusion and Valia-Chien cells, Ussing chambers and perfusion chambers as well as whole eye models. ^16^ These ex vivo models can be expensive both in terms of equipment and because they require a similar number of animals to in vivo models and large amounts of initial compound and tissue^12,17^.

Previously reported in vitro models take significant time to culture cell layers to establish the model and suffer from variable levels of epithelial resistance with compromised barrier function and therefore variable permeability, making these models less suitable for drug penetration studies^16,17^.

To be consistent with the 3 Rs of Reduction, Refinement and Replacement in animal experimentation, there is therefore a need for a model that replaces aspects of in vivo testing and, in contrast to existing ex vivo models, reduces the number of animals required for experiments. In addition to reducing the number of animals required, a further benefit of an ex vivo model that is able to use tissue from one eye in multiple tests is that more data can be derived from each human tissue sample, maximising the utility of this scare resource.

We therefore aimed to: (1), develop an ex vivo model to assess the penetration of topically applied molecules through ocular tissue and; (2), assess the penetration of siCASP2 through ocular tissue. We used ex vivo porcine cornea (similar biomechanical properties to human cornea)^18^ and human tissue in which corneal and human scleral penetration was assessed in 5 mm corneal disc explants, allowing the use of multiple explants per eye in a higher throughput assay than previous systems.

## Methods

### Corneal penetration model

#### Porcine cornea

**Figure 1:**
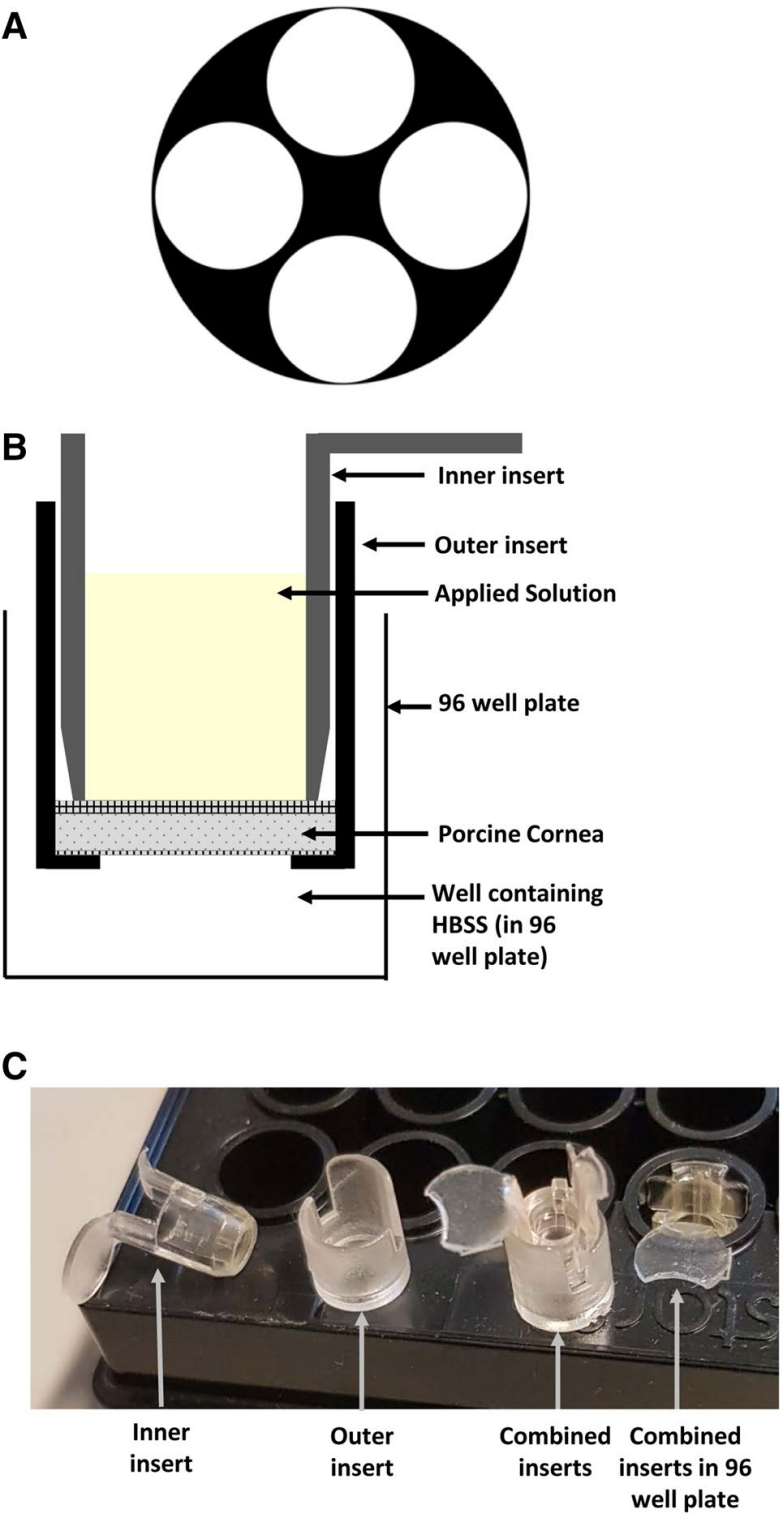
Corneal model for measuring penetration. (**A**) Illustrative diagram of the location from which 5 mm discs were cut from the cornea. (**B**) Illustrative diagram showing a 5 mm porcine corneal disc fitted between plastic 96 well plate inserts. The combined insert is placed into a well containing Hanks’ Balanced Salt Solution (HBSS). Solution containing the compound of interest is then applied to the upper, epithelial, surface shown in yellow. (**C**) Photograph of inserts showing inner insert and outer inserts separately. The disc of corneal tissue is placed in the base of the outer insert and the inner insert placed on top to sandwich it against the base before placing the combined inserts into the 96 well plate.

To assess corneal penetration we developed a 96 well plate format with fresh porcine cornea. Eyes were obtained from a local abattoir, tissues that were surplus in the food industry, within 2 h of death. Corneo-scleral discs were excised with dissecting scissors and placed epithelium-down in a petri dish containing Hank’s balance salt solution (HBSS; Invitrogen, Paisley, UK). 5 mm corneal discs were cut using a biopsy punch as shown in Fig. 1A (Stiefel, GSK, Middlesex, UK). Approximately 4 punches were taken from each cornea and fitted into a CellCrown™ 96 well insert (Sigma, Poole, UK), made of medical grade polycarbonate. The 5 mm corneal discs were placed into the base of the outer insert (Fig. 1B, C) with the epithelium facing up. The inner insert (Fig. 1B, C) was then placed inside the outer insert and on top of the corneal disc with sufficient pressure to create a water-tight seal but not enough to deform the cornea. The inserts were placed into wells in a 96 well plate containing 100 µl of HBSS (Invitrogen, Paisley, UK) and covered with parafilm to prevent evaporation.

To assess substances of varying penetrance, 30 µl of study solution were pipetted onto the epithelial surface with n ≥ 3 wells/treatment (Fig. 1B, C). The substances added were: HBSS (control), 1% rhodamine B, 1% fluorescein, 1 M sodium hydroxide (pH 12), 1% dextran blue (negative control), and 10 µg of siCASP2 (supplied by Quark Pharmaceuticals Inc, Israel). Plates were sealed with parafilm and placed in the incubator at 37 ± 1 °C and 5% CO_2_. After 30 min, 1 h, 2 h and 4 h, the inserts were transferred to new 96 well plates containing HBSS and the concentration in the remaining well was measured to assess trans-corneal penetration. The pH change in the endothelial medium induced by sodium hydroxide was assessed using pH strips. Rhodamine B (530 nm), fluorescein (490 nm) and dextran blue (610 nm) penetration were quantified using a microplate reader (Victor 3, Perkin Elmer). Each experiment was repeated on at least 3 independent occasions.

#### Human cornea

Human aspects of this study complied with the Declaration of Helsinki and its current amendments and was approved by the Dudley Local Research Ethics Committee (Ocular Surface Microenvironment in Health and Disease, REC reference number: 06/Q2702/44), including the determination that consent from legal representative was not required for the post-mortem human tissue use.

Donor cornea surplus to corneal transplant requirement after preparation for Descemet Stripping Automated Endothelial Keratoplasty (DSAEK) and penetrating keratoplasty (PK), was obtained from Birmingham and Midland Eye Centre, including discs of central lamellar cornea left after preparation for DSAEK and corneo-scleral rims left after preparation for PK. Discs of central cornea (without the endothelium; 5 mm disc), peripheral cornea (with endothelium; 4 mm disc) and sclera (5 mm) were prepared as for porcine tissue above.

### Haematoxylin and Eosin staining for corneal integrity

Tissues from the penetration experiments were fixed in 4% paraformaldehyde (Sigma) overnight. They were then cryopreserved in sucrose solutions at 10%, 20% and 30% for 6 h each and embedded in optimal cutting temperature (OCT) compound (Fisher Scientific, Loughborough, UK) and sectioned on a cryostat (Bright Instruments, Huntingdon, UK). In order to determine the integrity of the epithelial layers the slides were washed in 0.1 M phosphate buffered saline (PBS) and then stained with Harris Haematoxylin (Sigma), washed and differentiated in a 1% acid alcohol solution, washed in sodium bicarbonate for 2 min and finally counterstained in Eosin (Sigma). Sections were washed in tap water and dehydrated though a graded series of ethanols, cleared in Histoclear (National Diagnostics) and coverslips mounted using Vectamount (Vector Labs, Peterborough, UK) and then visualised using the Brightfield setting on an AxioPlan 2 microscope equipped with an AxioCam HRc camera and running Axiovision Software (all from Zeiss, Herfordshire, UK).

### Trans-epithelial resistance measurements

Trans-epithelial resistance (TER) was measured using the Millicell electrical resistance monitor (Millipore, Watford, UK), specifically designed for 96 well plate measurements, on separate corneal explants conducted in parallel to penetration experiments. Measurements were taken of HBSS only wells for background, cornea treated with HBSS and cornea treated with pH 12 NaOH. Resistance data were collected at 0, 30, 60, 120 and 240 min. Background values were subtracted from resistance measurements and the data was corrected for the area covered by the epithelial layer (5 mm in diameter with an area of 0.1963 cm^2^). Experiments were repeated three times.

### siCASP2 sequence and source

Chemically modified siRNA against caspase-2 (siCASP2) (supplied by Quark Pharmaceuticals Inc, Israel) was produced as previously described and had been chemically stabilized by Quark laboratories with the following sequence 5′-GCCAGAAUGUGGAACUCCU-3′ (sense strand)^19^. siCASP2 was chosen because this is a siRNA therapy currently in clinical trial (www.eyeactnow.org).

### siCASP 2 delivery

10 µg siCASP2 in 30 µl of HBSS either applied to the epithelial surface of the corneal explant and left on (continuous application) or applied and washed off with HBSS after 2 min (transient application) in order to approximate clearance by lacrimal drainage.

### siCASP2 analysis by qPCR

siRNA penetration and corneal adsorption were measured by qPCR methods as described previously^19^. siCASP2 levels were measured in the endothelial HBSS medium (to assess transcorneal penetration) and corneal explant tissue. We measured the rate of penetration at 30 min, 60 min, 120 min and 240 min. Corneal tissue was analysed at 60 min and 240 min and homogenised for RNA extraction using the RNeasy extraction kit (Qiagen, Manchester, UK). The RNA was then converted to cDNA using the Tetro cDNA synthesis kit (Bioline, London, UK) with the use of a predesigned stem and loop primer (5′-GTCGTATCCAGTGCAGGGT CCGAGGTATTCGCACTGGATACGACGCCAGA-3′)^20^ in place of those provided in the kit. siCASP2 levels were quantified using the standard curve qPCR method. The 7 point standard curve was generated using known quantities of siCASP2 in a SYBR green (Applied Biosystems, Warrington, UK) reaction with previously designed siCASP2 primers: forward- 5′GGCGGAGGAGTTCCACATTC-3′ and reverse-5′-GTGCAGGGTCCGAGGT-3′. Cornea treated with HBSS and the subsequent endothelial culture medium was used as a control.

### Fluorescence in situ hybridisation (FISH)

Cornea treated with HBSS and 10 µg of siCASP2 (continuous and transient application) were fixed and sectioned as described above. FISH was then carried out according to previously published methods with some modifications^21^. Sections were washed in PBS, permeabilised in 0.1% triton x-100 for 10 min and incubated for 20 min in 20% glycerol after which the slides underwent 3 cycles of freeze–thaw on dry ice. Slides were washed in PBS and incubated with 0.1 M HCL for 30 min at room temperature. After a PBS wash, 0.5% Triton x-100 was added for 30 min. Slides were washed in PBS and then equilibrated in 50% formamide made with 2 × saline-sodium citrate (SSC) buffer for 10 min. During equilibration the hybridisation solution was made consisting of 50% formamide, 2 × SSC buffer, 10% dextran sulphate, 300 ng/ml of salmon sperm DNA and 250 ng/ml of siCASP2 probe which corresponded to the sense strand of the siCASP2 sequence. This was pipetted onto the slides and a coverslip was applied. Slides were heated to 78 °C for 2 min on an inverted heating block after which they were incubated at 37 °C in a humidified chamber overnight. Slides were placed in 2 x SSC until the coverslips loosened and came off after which they were washed × 3 in 2 x SSC for 5 min. Sections were counter-stained with DAPI and coverslips applied. Slides were visualised using the Axioplan 2 epi-fluorescent microscope.

### Immunohistochemistry

After running the assay, corneal discs were removed and fixed overnight in 4% paraformaldehyde in phosphate-buffered saline then cryoprotected in a graded series of sucrose solutions and embedded in optimal cutting temperature (OCT) compound. Sections were cut perpendicular to the corneal surface at a thickness of 15 µm using a cryostat (Brights Instruments, Huntingdon, UK) and adhered onto SuperFrost™ (Fisher Scientific, Loughborough, UK) coated glass microscope slides and stored at − 80 °C until required. Frozen sections thawed for 20 min and washed 3 × 5 min in PBS before 20 min permeabilisation and blocking non-specific sites in PBS containing 1% Triton-X-100 (Sigma) and 3% bovine serum albumin (BSA; Sigma). Sections were incubated overnight at 4 °C with primary antibody against ZO-1 (Rabbit polyclonal, Invitrogen, Carlsbad, CA, USA) in 0.5% Tween-20 and 3% BSA before washing 3 × 5 min in PBS and incubating with secondary antibody (Alexa 488, goat anti-rabbit, Invitrogen) at room temperature (RT). Sections were washed 3 × 5 min in PBS then mounted in Vectashield mounting medium containing DAPI (Vector Laboratories, Peterborough, UK). Controls with omitted primary antibody were included in each run and were used to set the background threshold levels of fluoresence prior to image capture. Confocal microscopic images were captured on Zeiss LSM880 mp laser system (Zeiss).

### Statistical analysis

Data were analysed in SPSS 21 (IBM Corp., Armonk, NY USA) with generalised estimating equations used to model percentage penetration/adsorption. Data was considered to be significant when the probability of type 1 error was less than 0.05. Values are displayed as means with 95% confidence interval of the parameter estimate in square brackets. Permeability was calculated as previously described using the equation $P=\frac{m}{AtC}$ where m = mass of compound moving through cross-sectional area A in time t and C is the initial concentration applied to the epithelial surface^22^.

## Results

### Porcine corneal permeability

**Figure 2:**
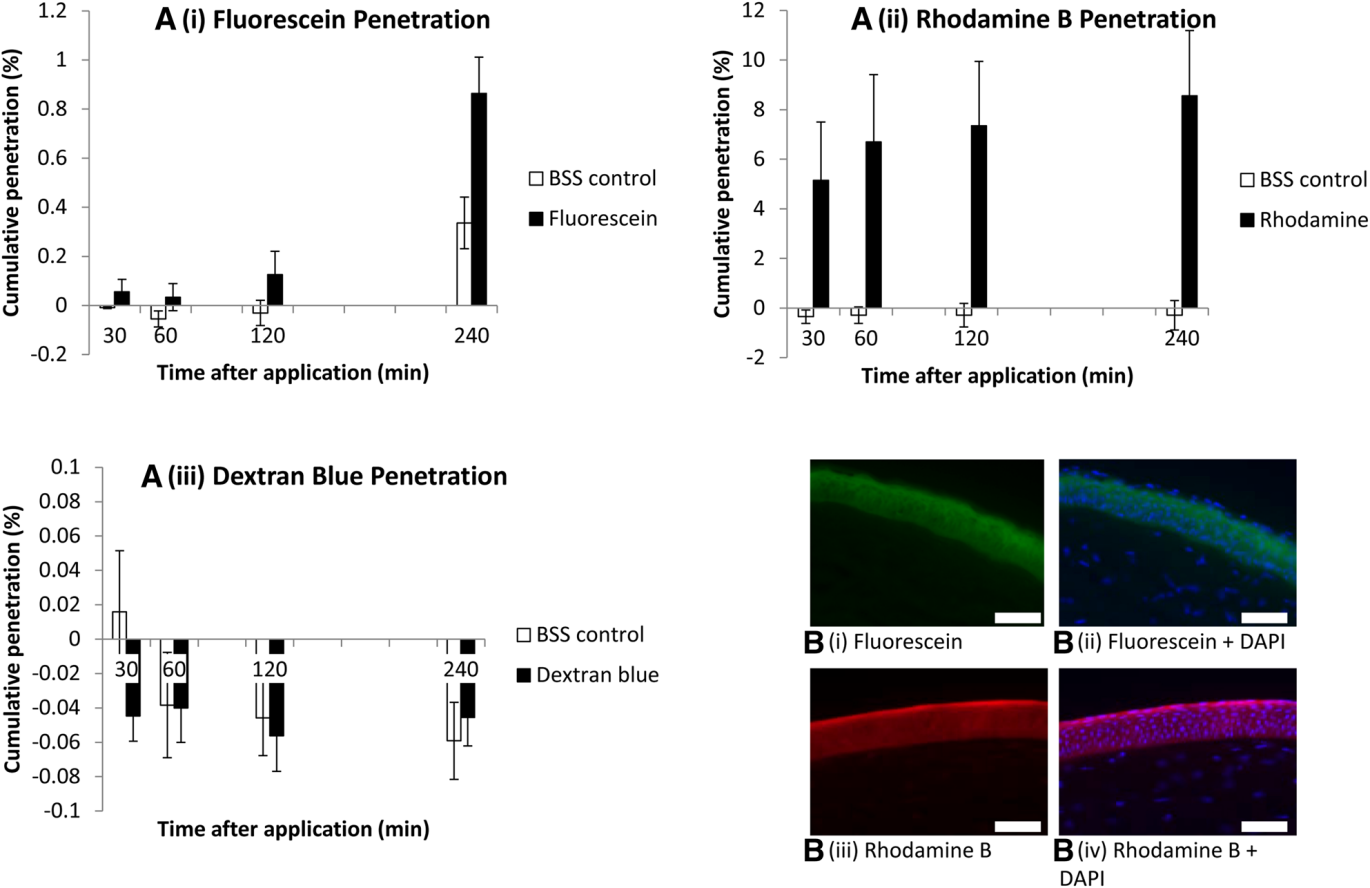
Ex vivo porcine corneal permeability to fluorescein (**A** i), rhodamine B (**A** ii) and dextran blue (**A** iii). Results are displayed as bar charts with mean and standard error (error bars). (**B** i–iv) fluorescent microscopy images of anterior stroma and epithelium after fluorescein (**B** i, ii) and rhodamine B (**B** iii, iv) application, without (**B** i, iii) and with (**B** ii, iv) DAPI staining of cell nuclei. Scale bar 100 µm. Graphs represents data from 3 independent experiments for rhodamine, 4 repeats for fluorescein and 5 repeats for dextran blue.

To determine whether our model was able to measure the penetration of compounds whilst allowing the cornea to retain its expected barrier function, we began by testing a series of reference compounds’ penetration. The cornea was minimally permeable to the hydrophilic fluorescein sodium (376 Da; penetration 0.089% [− 0.027 to 0.204] at 60 min; 0.528% [0.198–0.858] at 240 min; Fig. 2A i) with high levels of adsorption onto the corneal epithelium (Fig. 2B i, ii) and a calculated permeability coefficient of 7.76 × 10^−6^ cm/min [− 2.32 to 17.8]. In contrast, penetration of the hydrophobic rhodamine B (479 Da) was greater than that of fluorescein (penetration 6.98% [3.83–10.14] at 60 min; 8.85% [5.64–12.1] at 240 min; Fig. 2A ii) with a calculated permeability coefficient of 6.10 × 10^–4^ cm/min [3.34–8.86] and also adsorbed to the corneal epithelium (Fig. 2B iii, iv). The large hydrophilic molecule, dextran blue (2000 kDa), was used as a negative control to show that the cornea retained a barrier function and that no leakage around the insert occurred. Dextran blue should not penetrate the intact cornea and did not do so for the duration of the experiment (Fig. 2A iii).

### Porcine Corneal integrity

**Figure 3:**
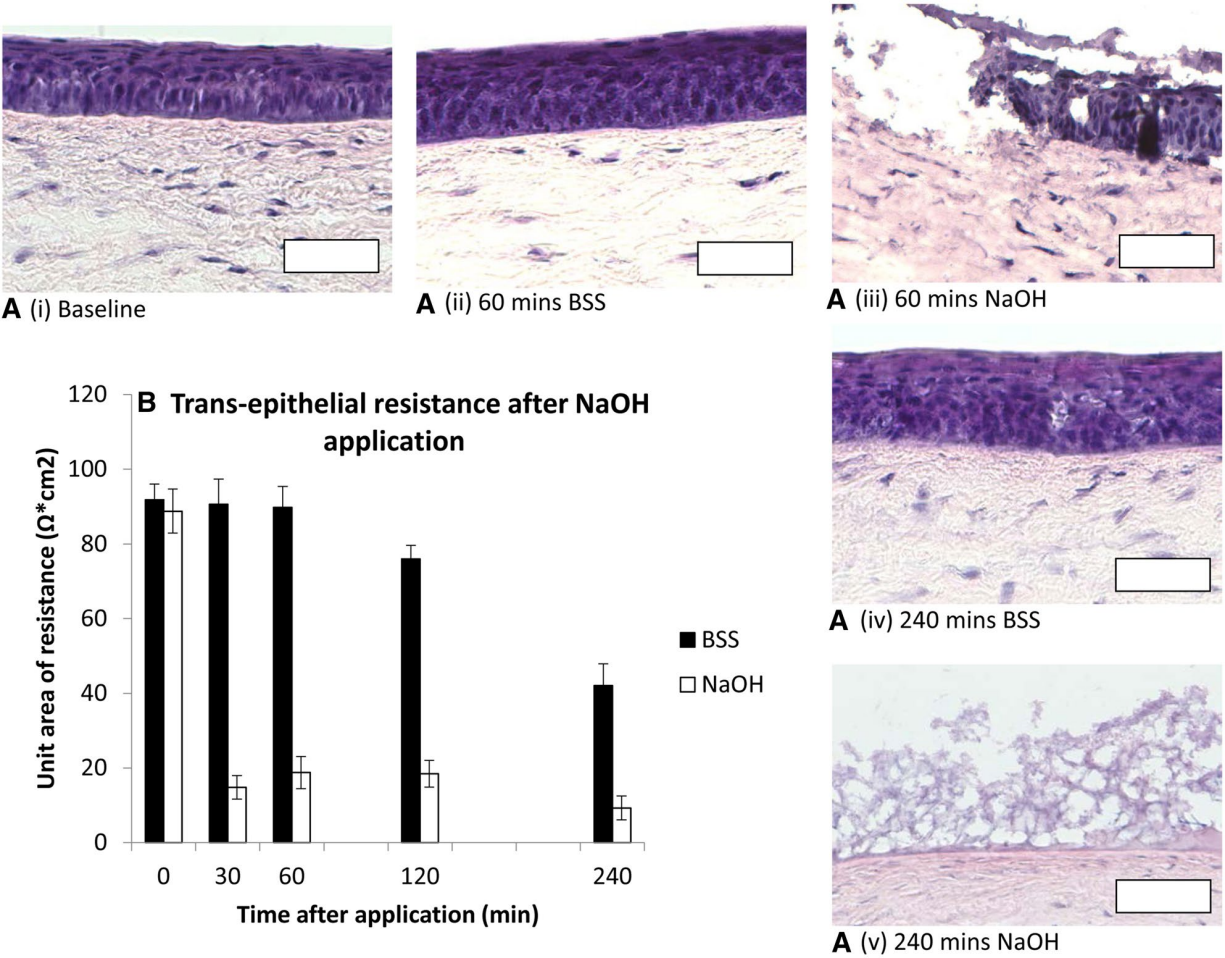
(**A**) H&E stained corneal epithelial histology. Epithelial structure remains intact 240 min after BSS application (i, ii, iv). NaOH destroyed epithelial architecture (iii, v). Scale bar 100 µm. (**B**) Transepithelial resistance (TER) displayed as bar charts with mean and standard error (error bars). TER was preserved until 60 min after BSS application. A small decrease in TER was evident 120 min after BSS application, with a substantial drop by 240 min. NaOH caused rapid loss of TER.

**Figure 4:**
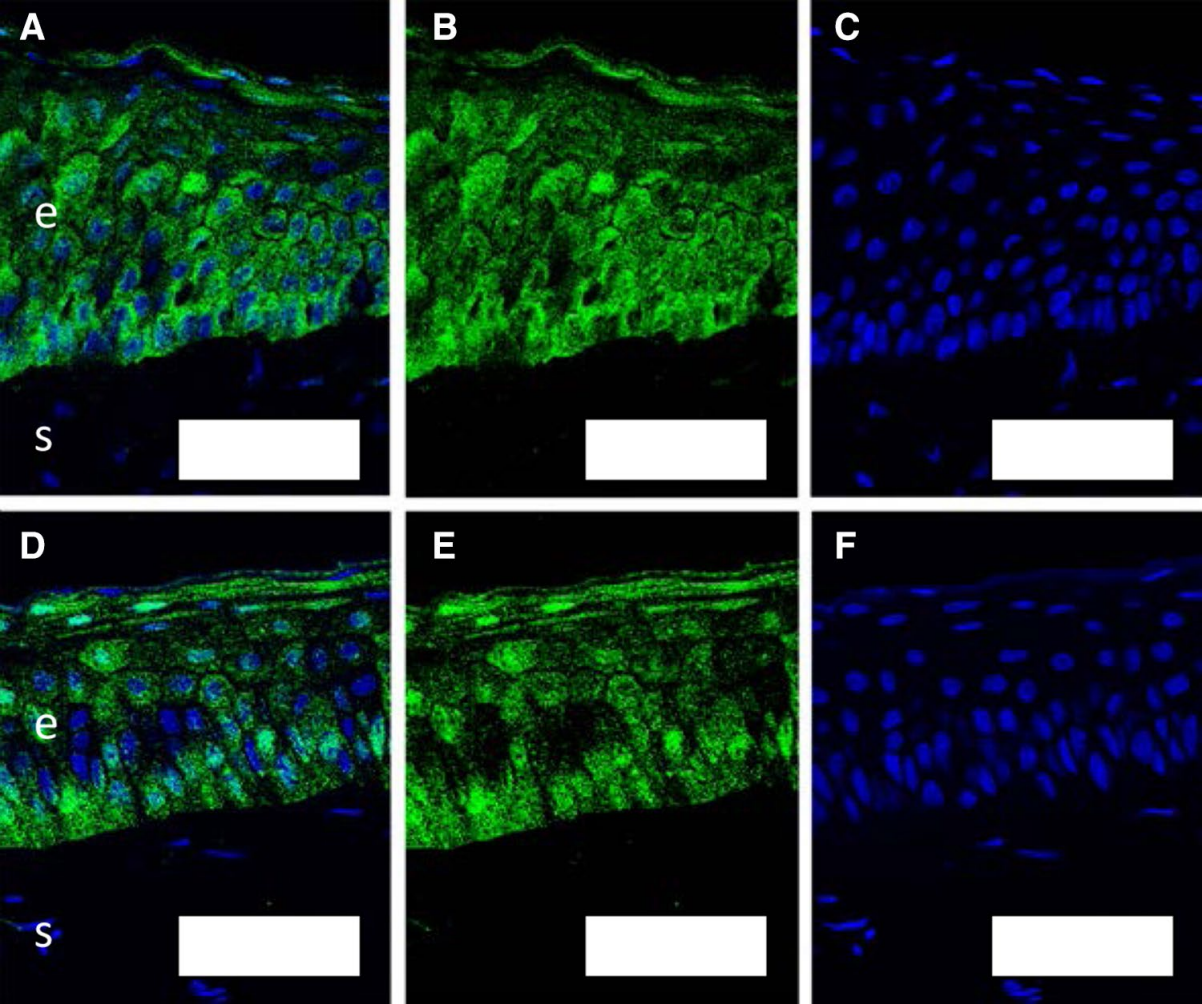
ZO-1 staining for epithelial tight junctions in corneal sections at baseline (**A**–**C**) and after 60 min (**D**–**F**) in the model with HBSS appled. (**A**, **D**) combined images; (**B**, **E**) ZO-1 staining in green; (**C**, **F**) DAPI-stained nuclei. “e” marks corneal epithelium; “s” marks corneal stroma; scale bar 50 µm.

The corneal epithelial structure, demonstrated on H&E staining was preserved over the first 60 min of the assay, with mild disruption of epithelial integrity by 240 min (Fig. 3.A i, ii and iv). The corneal epithelial unit area of resistance was stable after BSS application at 90 Ω cm^2^ [75–105] until 60 min, falling to 76 Ω cm^2^ [57–95] at 120 min and 42 Ω cm^2^ [33–52] at 240 min (Fig. 3B). Up to 60 min, ZO-1 antibodies demonstrated strong immunostaining for epithelial tight junctions (Fig. 4). After administration of NaOH as a positive control, pH in the endothelial culture medium increased to pH 8–9 at 30 min and TER decreased to 15 Ω cm^2^ [6–24] (Fig. 3B), with destruction of the corneal epithelium on H&E staining (Fig. 3A iii, v).

### Permeability of the cornea to siCASP2

**Figure 5:**
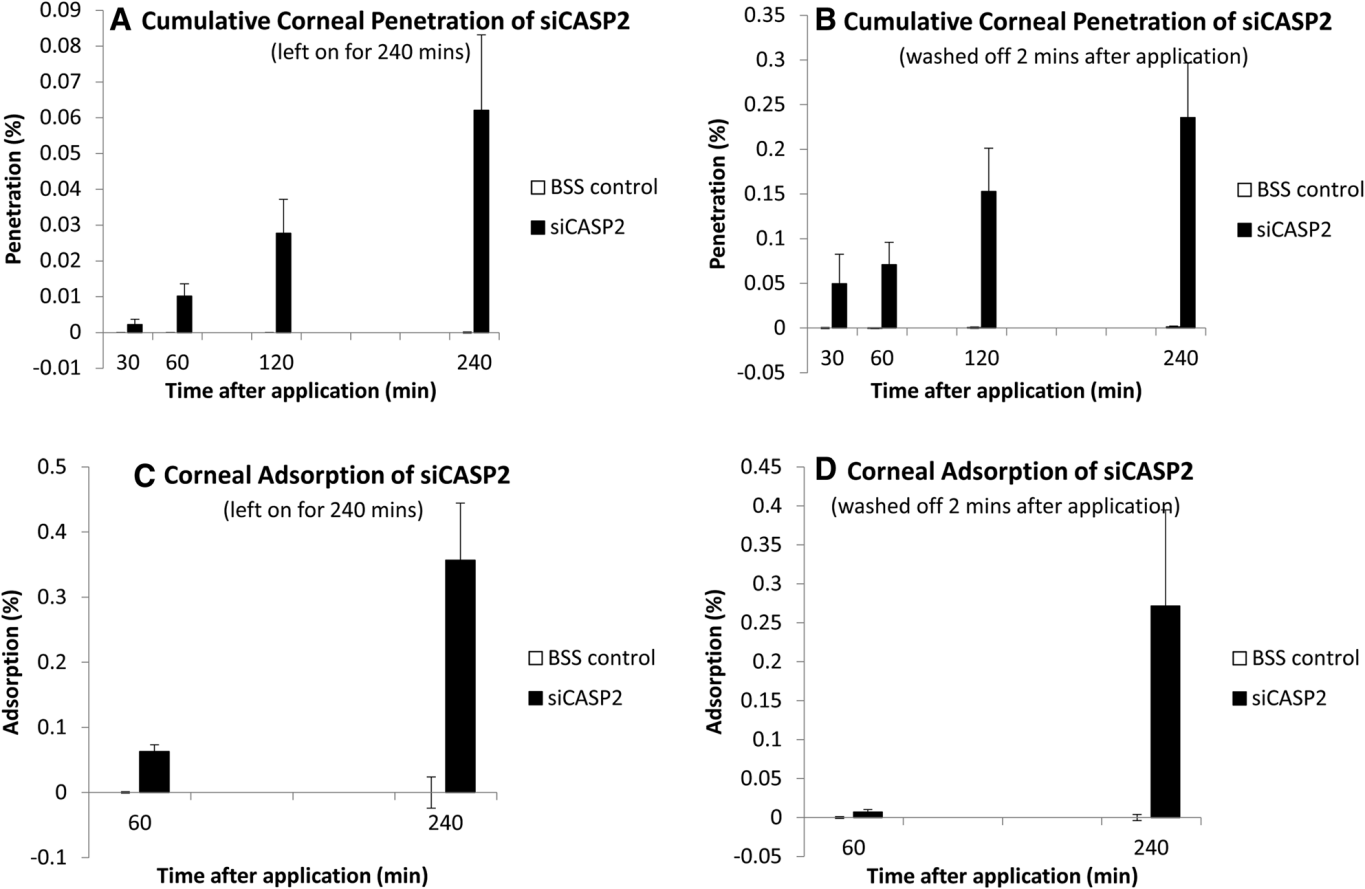
siCASP2 penetration through and adsorption to the cornea after siCASP2 was applied to the epithelial surface and left on for 240 min (**A**, **C**) or washed off after 2 min (**B**, **D**). Results are displayed as bar charts with mean and standard error (error bars). Graphs represents data from n = 4 independent experiments for penetration and n = 3 independent experiments for corneal adsorption.

After application of 10 µg of siCASP2 (hydrophilic, 12 kDa) to the corneal surface, 1.0 × 10^–2^% [0.49–1.54] penetrated by 60 min and 6.2 × 10^–2^% [2.28–10.1] by 240 min (Fig. 5A), giving a calculated permeability coefficient of 8.73 × 10^–7^ cm/min [1.06–3.36]. When the applied siCASP2 was washed off 2 min after application, to simulate lacrimal drainage after hypothetical eyedrop application, 7.10 × 10^–2^% [− 3.74–17.9] penetrated by 60 min and 0.234% [0.171–0.298] by 240 min (Fig. 5B).

### Corneal siCASP2 adsorption

We further investigated corneal siCASP2 adsorption at 60 and 240 min. When the applied siCASP2 was left on the corneal epithelial surface, 0.031% [ −  0.05 to 0.109] of the initial 10µ applied was adsorbed to the cornea by 60 min and 0.178% [0.124–0.233] by 240 min (Fig. 5C). When the applied siCASP2 was washed off after 2 min, 0.003% [− 0.08 to 0.09] was adsorbed to the cornea by 60 min and 0.075% [0.011–0.138] by 240 min (Fig. 5D).

**Figure 6:**
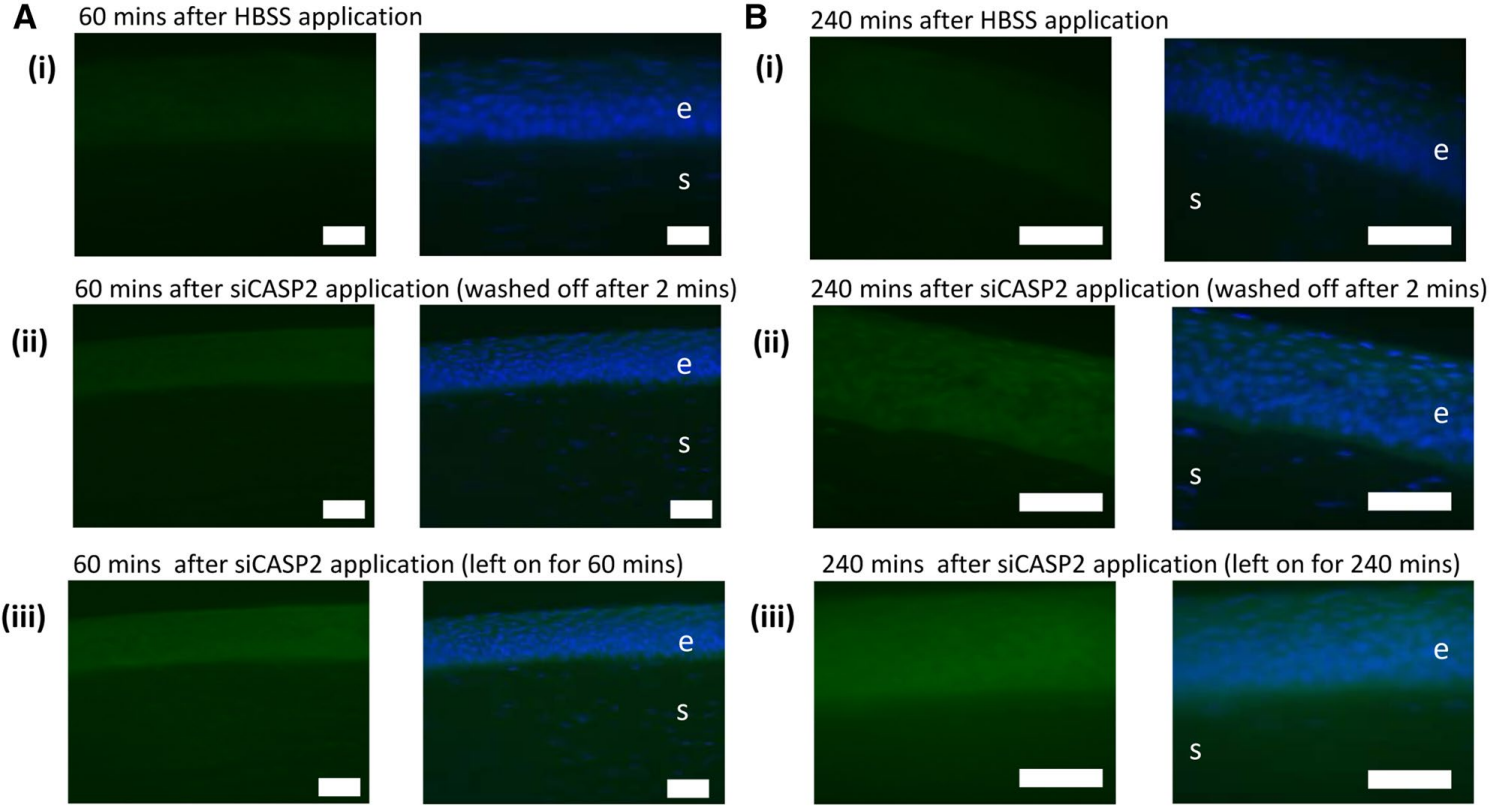
siCASP2 adsorption to the corneal epithelium demonstrated by FISH staining. 10 µg siCASP2 was applied to the corneal surface and left on for the duration of the experiment (**A**) or washed off after 2 min (**B**) to simulate lacrimal drainage. Green staining represents siCASP2 and blue represents DAPI (nucleic acid) staining. Background fluorescence is seen in (**A** i) and (**B** i) images of cornea 60 and 240 min after HBSS application. “e” marks corneal epithelium; “s” marks corneal stroma; scale bar 50 µm.

Fluorescence in situ hybridisation using a probe complimentary to the sense strand of siCASP2 localised the adsorbed siRNA to the corneal epithelium, but not the stroma, with qualitatively greater fluorescence at 240 compared to 60 min and when the siCASP2 was left on rather than washed off, consistent with the qPCR data (Fig. 6).

### SiCASP2 penetration and adsorption through human ocular tissues

**Figure 7:**
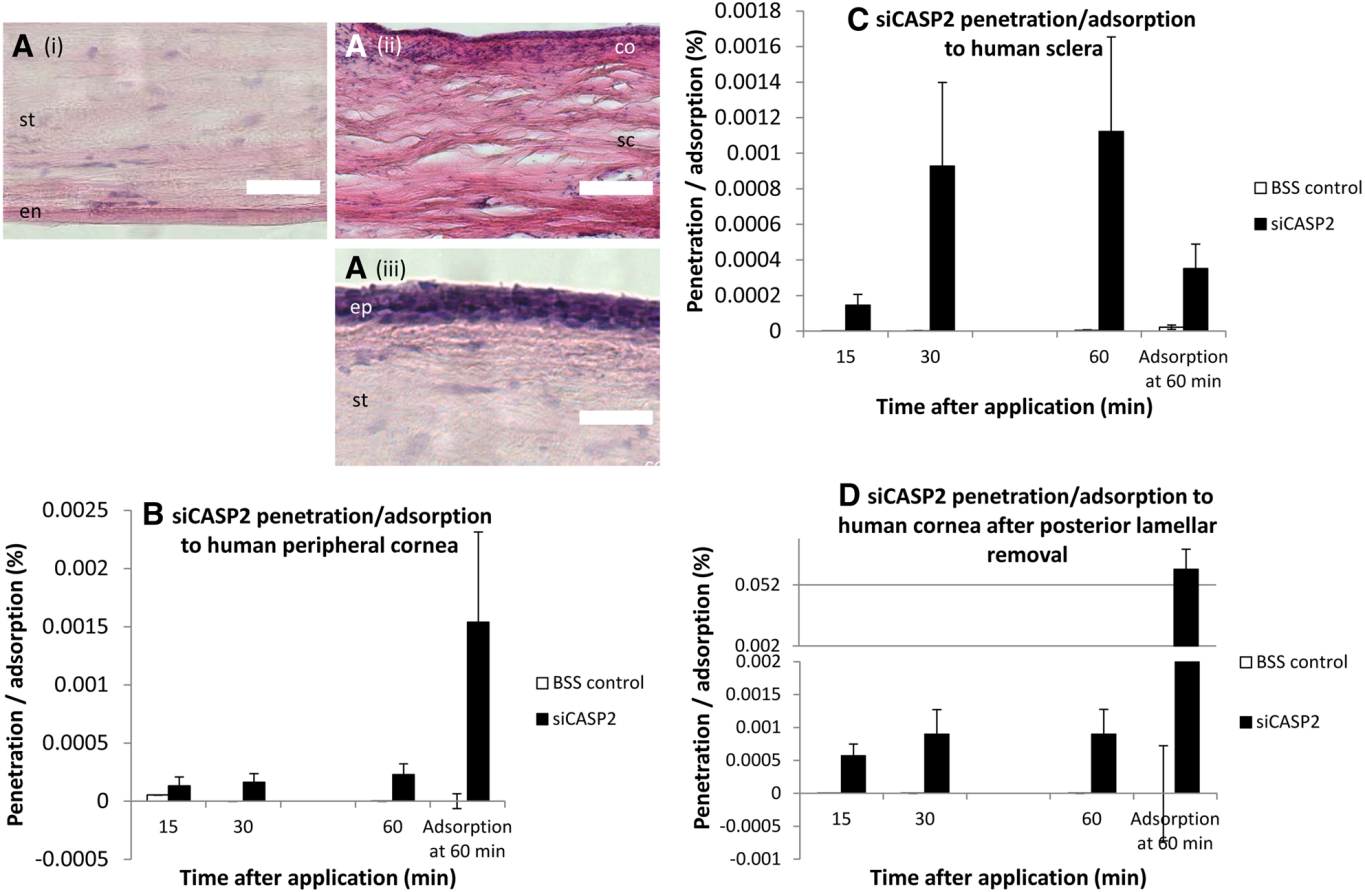
(**A**) representative H&E-stained human corneal tissue from the assays in (**B**–**D**): (i), human peripheral cornea; (ii), human sclera; (iii), human central cornea after posterior lamellae removal. (**B**–**D**) Penetration of siCASP2 through and adsorption to human cornea and sclera over 60 min after siCASP2 was applied to the epithelial surface and washed off after 2 min. Results are displayed as bar charts with mean and standard error (error bars). (**D**) is displayed with a split y axis. “ep” corneal epithelium; “s” corneal stroma; “en” corneal endothelium; “sc” sclera; “co” conjunctiva; scale bar 100 µm. (**B**, **C**) represent data from n = 2 independent experiments; (**D**) represents data from n = 3 independent experiments.

Because porcine TER suggested degradation of epithelial integrity after 60–120 min, we assessed penetration in human studies over a 60 min experiment. Because human corneal tissue is a very limited resource and TER measurements require duplicate tissue, we did not measure TER. Peripheral cornea and sclera from the donor corneoscleral rim displayed normal tissue structure (Fig. 7A i, ii). Surplus corneal tissue after preparation for DSAEK, from which the endothelium and inner stroma had been removed by microkeratome (Fig. 7A iii), displayed normal epithelial structure. siCASP2 was applied to the upper/epithelial surface for 2 min then washed off for all tissues, after which, 2.29 × 10^–4^% [0.64–3.95] siCASP2 penetrated peripheral cornea by 60 min and 1.54 × 10^–3^% [0.14–2.93] was adsorbed (Fig. 7B). Human sclera allowed comparable penetration (1.12 × 10^–3^% [0.17–2.07]) and adsorption (3.31 × 10^–4^% [0.84–5.77]; Fig. 7C). Cornea lacking the inner stroma (DSAEK donor tissue) had comparable penetration of 8.96 × 10^–4^% [3.93–13.9] of the applied siCASP2 but much higher levels of adsorption at 0.065% [0.037–0.093] (Fig. 7D). There was no evidence of a difference in siCASP2 penetration between porcine and human tissue (*p* = 0.71) or between cornea and sclera (*p* = 0.156).

## Discussion

We describe a novel, efficient, medium-throughput ex vivo model for assessing transcorneal drug penetration. Transcorneal penetration of the hydrophobic rhodamine B is higher than the hydrophillic sodium fluorescein and penetration of siRNA is low. siCASP2 penetrates the cornea and we did not find differences in transcorneal penetration of siRNA between porcine and human tissue or between human cornea and human sclera in the same model.

Previous ex vivo models include the use of Franz diffusion cells, which allow assessment of corneal permeablity using whole corneal explants^23,24^. By assessing penetration across 5 mm discs, our model allows a greater number of assays to be performed per eye and comparison of multiple conditions in tissue from the same eye.

As expected, porcine cornea was impermeable to the negative control solution of dextran blue, which has a molecular weight of 2,000 kDA, far exceeding the highest reported size limit for corneal permeability of 500 Da^25,26^. The cornea displayed low levels of permeability to the hydrophilic sodium fluorescein and greater permeability to the hydrophobic rhodamine B, with calculated permeabilities comparable to previous reports using whole corneal preparations, which have reported for instance that the permeability to rhodamine permeability was 3.72 × 10^–4^ cm/min (compared to our finding of 6.10 × 10^4^ cm/min) and fluorescein was 4.21 × 10^–5^ cm/min ex vivo and 1.62 × 10^–5^ in vivo (compared to our finding of 7.76 × 10^–6^ cm/min)^27,28^. The variability in measurement of fluorescein concentration was greater than that for both rhodamine and siCASP2, reflecting the limitations of direct fluorescein measurement at very low concentrations. Rhodamine B and fluorescein were most adherent to the corneal epithelium on fluorescence microscopy, also consistent with previous reports studying intact cornea^24,29–31^.

The epithelium forms the main barrier restricting drug penetration^10^, and we therefore demonstrated corneal epithelial integrity on histology, showing an intact corneal epithelial layer after 240 min, in accordance with previous publications that have demonstrated the integrity of the cornea over greater periods of time^31–33^. However, on our sections at 4 h, there was some mild loss of structural integrity by 240 min and histology cannot quantify the functionality of epithelial tight junctions, breakdown of which could increase penetration. We also measured electrical resistance of the epithelium to an alternating current, which suggested that epithelial resistance decreased after 2 h within the assay (4 h post-mortem). We therefore considered measurements taken before the 120 min time point to be most relevant to the living eye.

Post transcriptional gene silencing by siRNA is a promising treatment^1,34^. siRNA treatments are more cost effective and more easily synthesised then inhibitory proteins and allow for increased specificity. siCASP2 is at the forefront of ocular siRNA treatment with data demonstrating increased RGC survival after intravitreal siCASP2 treatment^3,4^, and one phase III trial of siCASP2 ongoing to treat non-arteritic anterior ischaemic optic neuropathy, delivered by intravitreal injection (NCT02341560; ClinicalTrials.gov). We therefore intended to assess ocular penetration of siCASP2 as an index siRNA.

The corneal permeability of siCASP2 was low, but comparable to that of fluorescein, despite being 30 × larger (376 vs 12,000 Da) and well above the reported size limits for corneal permeability^25,26^. siCASP2 is therefore the largest molecule that has been reported to penetrate the cornea. In addition siRNA also have net negative charge and positive charge has been associated with improved ocular drug penetration from prolonged ocular surface contact time^35–37^.

The adsorption of siCASP2 to the epithelium (or to the plastic insert) may explain some of the continued increase in penetration from 30 to 240 min after the applied siCASP2 was washed off (2 min after application) and the observed increase in corneal adsorption after the 60 min timepoint. It may therefore be that the negative charge or other properties of siRNA facilitate some surface interaction and persistence of the siRNA. Variability in the assay results, especially for fluorescein and siCASP2 penetration, may reflect variability in tissue properties or the magnified effect of measurement variability when determining low concentrations. In addition, the assay differs from the in vivo environment in the lack of plasma proteins in ocular surface fluids.

The assay was versatile, allowing us to use waste human corneal tissue and assess ocular penetration through human central and peripheral cornea as well as human scleral tissue. Levels of siCASP2 penetration through central corneal tissue from which the posterior lamellae had been dissected were similar to full thickness peripheral cornea (non-significantly higher), consistent with the epithelium forming the main barrier to drug penetration^10^. The comparable penetration across sclera likely therefore also reflects the presence of the conjunctival epithelium. However, the greater adsorption to central cornea than peripheral cornea may reflect some degree of epithelial dysfunction following graft preparation^30^. We did not have sufficient tissue to measure TER on human cornea, so it may be that the human donor transplant tissue would have abnormal TER, because it is longer post-mortem than the porcine cornea. However, in porcine tissue we observed increased penetration associated with reduced TER (at 240 min), which was not the case in human tissue.

Whilst siCASP2 did penetrate the cornea more than might be expected based in its size, hydrophilicity and charge, corneal permeability to siCASP2 was still two orders of magnitude less than the 3.7 × 10^–5^ cm/s reported for dexamethasone acetate^10^, a drug with good anterior segment penetration. Therefore, in order to achieve biologically relevant retinal concentrations of siRNA in the anterior or posterior segments, future work should consider penetration-enhancing adjuncts to enhance penetration.

Corneal epithelial cells express multiple species-specific influx and efflux transporters, which affect drug penetration such as the efflux proteins including the adenosine triphosphate binding cassette g2 protein (also known as the breast cancer resistance protein) found in human but not porcine or rabbit cornea and P-glycoprotein (also known as multidrug resistance protein 1)^14^, which is expressed in normal rabbit (but not human or porcine) corneal epithelium, where its inhibition increases corneal drug penetration^14,38^. Influx transporters are likely also to play are role in transcorneal permeability of the substances tested and while nucleic acid and single nucleoside transporters are present, influx transporters are less well studied in the cornea than efflux transporters^14,39^.

We have reported and validated a novel medium-throughput ex vivo model to assess transcorneal drug delivery, which allowed assessment of the corneal penetration of siCASP2. siCASP2 penetrated the cornea and sclera, despite its large molecular weight and negative charge, but at lower levels than other drugs used to treat anterior segment pathology, suggesting that continued intra-ocular administration of siRNA-based therapy will be necessary unless penetration can be improved with carrier molecules.

## Data Availability

The datasets generated during and/or analysed during the current study are available from the corresponding author on reasonable request.
